# The European challenges of funding orphan medicinal products

**DOI:** 10.1186/s13023-018-0927-y

**Published:** 2018-11-06

**Authors:** Márta Szegedi, Tamás Zelei, Francis Arickx, Anna Bucsics, Emanuelle Cohn-Zanchetta, Jurij Fürst, Maria Kamusheva, Pawel Kawalec, Guenka Petrova, Juraj Slaby, Ewa Stawowczyk, Milan Vocelka, Ingrid Zechmeister-Koss, Zoltán Kaló, Mária Judit Molnár

**Affiliations:** 1 Institute of Genomic Medicine and Rare Disorders, Semmelweis University, H 1083 Tömő u, Budapest, 25-29 Hungary; 2 Department of Health Policy and Health Economics, Institute of Economics, Faculty of Social Sciences, Eötvös Loránd University, Pázmány Péter sétány 1/A 1117, Budapest, 361 Hungary; 3 Syreon Research Institute, Mexikói út 65A, Budapest, 1142 Hungary; 4 National Institute for Health and Disability Insurance of Belgium, Av. de Tervuerenlaan, 211, 1150 Brussels, Belgium; 5 Mechanism of Coordinated Access to Orphan Medicinal Products (MoCA), 1150 Vienna, Austria; 6 French National Authority for Health, 5, avenue du Stade de France –, 93218 Saint-Denis, La Plaine Cedex France; 7 Health Insurance Institute of Slovenia, Miklošičeva cesta 24, 1507 Ljubljana, Slovenia; 8 Department of Organization and Economics of Pharmacy, Medical University, Sofia, Bulgaria, Dunav str. 2, 1000 Sofia, Bulgaria; 9 Institute of Public Health, Faculty of Health Sciences, Jagellonian University, Medical College, 008 Grzegorzecka 20, 31-531 Kraków, Poland; 10 State Institute for Drug Control, Šrobárova 48, 100 41, 10 Praha, Czech Republic; 11 Department of Health, Economics, Ludwig Boltzmann Institute, Garnisongasse 7/20, A-1090 Vienna, Austria

**Keywords:** Orphan medicinal products, Funding, Reimbursement, Patient access, Equity, European Union

## Abstract

**Background:**

Funding of orphan medicinal products (OMPs) is an increasing challenge in the European Union (EU).

**Objectives:**

To identify the different methods for public funding of OMPs in order to map the availability for rare disease patients, as well as to compare the public expenditures on OMPs in 8 EU member states.

**Methods:**

Information on the reimbursement status of 83 OMPs was collected in 8 countries by distinguishing standard and special reimbursements. In two consecutive years, the total public expenditures on OMPs were calculated by using annual EUR exchange rates. Annual total public expenditures were calculated per capita, and as a proportion of GDP, total public pharmaceutical and healthcare budgets. Differences between countries were compared by calculating the deviations from the average spending of countries.

**Results:**

In 2015 29.4–92.8% of the 83 OMPs were available with any kind of public reimbursement in participant countries including special reimbursement on an individual basis. In Austria, Belgium and France more OMPs were accessible for patients with public reimbursement than in Bulgaria, Czech Republic, Hungary and Poland. Standard reimbursement through retail pharmacies and/or hospitals was applied from 0 to 41% of OMPs. The average annual total public expenditure ranged between 1.4–23.5 €/capita in 2013 and 2014. Higher income countries spent more OMPs in absolute terms. Participant countries spent 0.018–0.066% of their GDPs on funding OMPs. Average expenditures on OMPs were ranged between 2.25–6.51% of the public pharmaceutical budget, and 0.44–0.96% of public healthcare expenditures.

**Conclusions:**

Standard and special reimbursement techniques play different roles in participant countries. The number of accessible OMPs indicated an equity gap between Eastern and Western Europe. The spending on OMPs as a proportion of GDP, public pharmaceutical and healthcare expenditure was not higher in lower income countries, which indicates substantial differences in patient access to OMPs in favour of higher-income countries. Equity in access for patients with rare diseases is an important policy objective in each member state of the EU; however, equity in access should be harmonized at the European level.

**Electronic supplementary material:**

The online version of this article (10.1186/s13023-018-0927-y) contains supplementary material, which is available to authorized users.

## Background

Prior to public reimbursement of pharmaceuticals and medical devices, cost-effectiveness and budget impact are increasingly applied evaluation criteria alongside other conditions. Pharmaceutical manufacturers tend to increase prices of truly innovative new medicines. Reacting to the increasing health expenditures more and more third-party payers tend to rationalize their expenditures by implementing cost-effectiveness criterion. There is significant tension between manufacturers and payers in judging the economically justifiable price and this tension is even more expressive in case of orphan medicinal products (OMPs) for patients with rare diseases [1, 2]*.*

A developed and morally matured society should judge the value of therapeutic improvements without taking into account the rarity of diseases or the opportunity cost of public spending on new medicines. Objective decision-making for public reimbursement has to be based on clinical, economic and social criteria, considering the appropriateness and uncertainty of evidence [3–7].

Internationally accepted definitions for rare diseases (RDs) and for OMPs have not been harmonised yet, but based on the prevalence of diseases different approaches tend to be quite similar. According to the current definition of the European Union (EU), the RDs are mostly inherited life-threatening or chronically debilitating diseases, which affect fewer than 5 out of 10,000 people. Approximately, 5–8000 RDs and ailments have been diagnosed by the medical science [8]*.*

OMPs are indicated for the diagnosis, treatment or prevention of life-threatening or very serious conditions of patients with RDs [9]*.* The purpose of the legislation was to determine the qualitative criteria of orphan designation. Furthermore, the Regulation (EC) No. 141/2000 describes the incentives for research, development, and marketing authorization of medicines/methods intended for diagnostics, treatment or prevention of RDs [10]*.* European (or other international) inventories for OMPs are available on the European Medicines Agency (EMA) website and official European public assessment reports (EPAR) [11]. Orphanet is a reference portal for information on RDs and OMPs for all audiences. On this website, the orphan designations, as well as the OMPs authorized by different procedures from various countries (i.e. EU, Japan, and USA) are listed with related and relevant information [12]*.*

Usually, the prices of OMPs are significantly higher than pharmaceutical prices in common diseases. Health economic evaluation of OMPs is complicated due to difficulties in selecting policy relevant comparators, wide confidence intervals of efficacy parameters and serious adverse events, the lack of hard clinical endpoints in clinical trials and uncertainty in patient numbers and resource utilisation and treatment costs per patient. It is difficult to measure the efficacy and cost-effectiveness of OMPs; however, several proposals address this challenge [13–15]. From another point of view, the willingness to pay for one unit of health gain might be different for technologies in RDs; therefore, implementation of transparent criteria for pricing and reimbursement is a big challenge [16–18]*.*

In almost all EU countries, regulators, payers and healthcare providers should make additional efforts to improve the accessibility of patients with RDs to OMPs by special policy interventions and agreements [19, 20]. However, unaffordable prices and increasing expenditure on OMPs challenge the sustainability of healthcare funding in all countries [21]*.*

Previous studies concluded that external price referencing system prevented lower-income Central and Eastern European (CEE) countries from implementing value based pharmaceutical prices [22]. While the public healthcare budgets in lower-income countries are significantly lower, relatively higher OM prices induce greater burden to reimburse these medicines in CEE [23, 24]*.*

Our objective was to draw a map on the economic burden of 83 medicines with designated OM status in 2015 by EMA in 8 EU countries with different economic status and population size, including Austria, Belgium, Bulgaria, Czech Republic, France, Hungary, Poland and Slovenia. We investigated two aspects of patient access in countries with different economic status, the availability of OMPs with public reimbursement in 2015, and the public expenditure on OMPs in 2013 and 2014. We analysed whether public reimbursement is an increased challenge for lower-income countries.

## Methods

We found 83 medicines based on the list OMPs published at the Orphanet website and validated the list based on the pharmaceutical database of EMA [25, 26]. Competent authorities or institutes in the 8 participant countries were contacted to provide reimbursement status of these OMPs with specific details on the applied reimbursement technique in 2015. We intended to collect data from 8 countries with various population size and different geographical and economic status across the EU.

We evaluated reimbursement status in five categories, including (1) standard reimbursement through both retail pharmacies and hospitals; (2) standard reimbursement through retail pharmacies; (3) standard reimbursement in hospitals only; (4) special patient level reimbursement that is not automated based on patient eligibility but on individual requests; and (5) no public reimbursement.

The qualitative (reimbursement techniques) and quantitative data (total public expenditures - pharmaceutical and healthcare budgets) were provided by the national public administration bodies summarized in Table 1. Demographic and economic data (size of population, exchange rates, GDP) was obtained from the Eurostat website as the footnotes of the tables show. Austrian demographic data was not available in the Eurostat database, the source was the OECD website.

**Table 1:** The sources of the data per Member State

Austria	Federation of Austrian Social Insurance Institutions (Hauptverband der österreichischen Sozialversicherungsträger)
Belgium	National Institute for Health and Disability Insurance of Belgium (Institut National d’Assurance Maladie-Invalidité / Rijksinstituut voor ziekte-en invaliditeitsverzekering, INAMI / RIZIV)
Bulgaria	National Health Insurance Fund (NHIF) National Council on Prices and Reimbursement of Medicinal Products (NCPR)
Czech Republic	State Institute for Drug Control (Státní ústav pro kontrolu léčiv, SUKL)
France	French National Authority for Health (Haute Autorité de Sante, HAS)
Hungary	National Health Insurance Fund Administration of Hungary (Országos Egészségbiztosítási Pénztár, OEP)
Poland	National Health Fund (Narodowy Fundusz Zdrowia), Ministry of Health (Ministerstwo Zdrowia)
Slovenia	Health Insurance Institute of Slovenia (Zavod za zdravstveno zavarovanje Slovenije, ZZZS)

We also collected country level data on public OM spending in 2013 and 2014. Annual average total public expenditures in 2013–14 were calculated per capita, and as a proportion of GDP, total public pharmaceutical and healthcare budgets. We converted spending to Euros by applying the annual currency exchange rates based on Eurostat data.

We compared public expenditure on ten specific OMPs per 100.000 inhabitants in 2013 and 2014. We intended to select a representative sample of OMPs based on different attributes as field of the indication, existing therapeutic alternative, relative effectiveness (potentially curative/non-curative treatment), rarity (orphan/ ultra-orphan status) and cost commitment, as the Table 2 shows. A heterogeneous group of OMPs was collected, including idursulfase for mucopolysaccharidosis type II., rifunamide for Lennox–Gastaut syndrome, romiplostim for idiopathic thrombocytopenic purpura, trabectedin for sarcomas and ovarian neoplasms, nelarabine for special types of leukaemia or lymphoma, sildenafil for pulmonary hypertension, alglucosidase alfa for glycogen storage disease type II, icatibant for inadequate or non-functioning C1-Inhibitor protein and sapropterin for phenylketonurias, and eculizumab for paroxysmal nocturnal haemoglobinuria or for atypical haemolytic uremic syndrome.

**Table 2:** The attributes for selection of 10 Orphan Medicinal Products

	Field of the indication^a^	Other therapeutic alternative(s)?^a^	Clinical/Relative Effectiveness (potentially curative/non-curative treatment)^a^	Rarity (orphan/ ultra-orphan status)^a^	Cost commitment^b^
Alglucosidase alfa	Glycogen storage disease type II	No	Incremental (non-curative)	UO	Medium
Eculizumab	Paroxysmal nocturnal haemoglobinuria or for atypical haemolytic uremic syndrome	No	Major (non-curative)	O	High
Icatibant	Hereditary angioedema	Yes	Major (non-curative)	O	Medium
Idursulfase	Mucopolysaccharido-sis type II	No	Incremental (non-curative)	UO	High
Nelarabine	Special types of leukaemia or lymphoma	No	Curative	O	Medium
Rufinamide	Lennox–Gastaut syndrome	Yes	Major (non-curative)	O	Low
Romiplostim	Idiopathic thrombocytopenic purpura	No	Curative	O	Medium
Sapropterin	Phenylketonuria	Yes	Major (non-curative)	O	Low
Sildenafil	Pulmonary hypertension	No	Major (non-curative)	O	Low
Trabectedin	Sarcomas and ovarian neoplasms	No	Incremental (non-curative)	O	High

Abbreviations: *O* Orphan Medicinal Product, *UO* Ultra-orphan medicinal product

Sources: ^a^ The European public assessment reports (EPAR) for human medicines published by the European Medicines Agency (EMA) http://www.ema.europa.eu/ema/

^b^National Health Insurance Fund Administration of Hungary (Országos Egészségbiztosítási Pénztár, OEP)

## Results

**Fig. 1:**
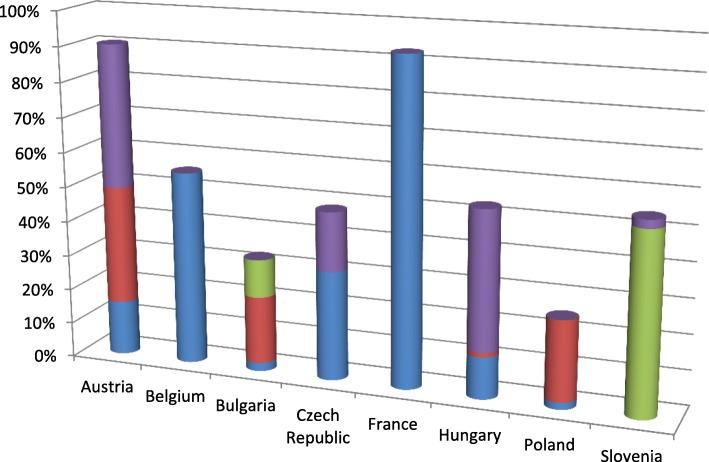
Reimbursement Status of Orphan Medicines in 8 EU Member States in 2015

In 2015, 29.4–92.8% of the 83 OMPs were available with any kind of public reimbursement in participant countries including special reimbursement on individual basis (see Fig. 1). The standard reimbursement listing was ranged from 0 to 41%.

Within the standard reimbursement techniques we distinguished reimbursement for outpatient care through retail pharmacies or inpatient care through hospitals.

Patients in Austria and France had over 90% access rate to OMPs, in Bulgaria and Poland patients had access to less than 30% of the 83 OMPs. The details of public reimbursement status of OMPs in 2015 are presented in the Additional file 1.

Data on public expenditure of OMPs was not accessible in France. In the 7 participant countries total public expenditures on OMPs were increased from 1.13–21.95 €/capita (mean: 7.36 €/capita) in 2013 to 1.69–25.04 €/capita (mean: 8.66 €/capita) in 2014. The average spending per capita in 2013–14 was ranged between1.41–23.50 € (mean: 8.63 €/capita). The absolute spending per capita showed 16.7 fold differences between countries with the highest and lowest spending. However, it should be noted that no data were available on hospital expenditure of OMPs from Austria and Bulgaria. Results are summarized in Table 3.

**Table 3:** The Total Public Expenditure on OMPs in Euro per Capita in 7 EU Countries in 2013 and 2014

	Austria^l^	Belgium	Bulgaria^m^	Czech Republic^n^	Hungary	Poland	Slovenia
2013							
Total public expenditure on OMPs in local currency	98,600,000 €^a^	245,000,000 €^b^	16,201,220 BGN^c^	1,100,000,000 CZK^d^	9,577,605,323 HUF^e^	212,725,536 PLN^f^	16,893,308 €^g^
Eurostat exchange rate - annual data (/1 €)			1.96 BGN^h^	25.98 CZK^h^	296.87 HUF^h^	4.20 PLN^h^	
Total public expenditure on OMPs in €	98,600,000	245,000,000	8266,000	42,340,000	32,262,000	50,649,000	16,893,000
Size of population	8,500,000^j^	11,161,642^i^	7,284,552^i^	10,516,125^i^	9,908,798^i^	38,062,535^i^	2,058,821^i^
Total public expenditure on OMPs in €/capita	11.60	21.95	1.13	4.03	3.26	1.33	8.21
2014							
Total public expenditure on OMPs in local currency	109,800,000 €^a^	280,000,000 €^b^	23,967,183 BGN^c^	1,400,000,000 CZK^o^	12,501,994,171 HUF^e^	348,368,792PLN^f^	19,853,716 €^g^
Eurostat exchange rate - annual data (/1 €)			1.96 BGN^h^	27.54 CZK^h^	308.71 HUF^h^	4.18 PLN^h^	
Total public expenditure on OMPs in €	109,800,000	280,000,000	12,228,000	50,835,000	40,496,000	83,342,000	19,854,000
Size of population	8,500,000^k^	11,180,840^i^	7,245,677^i^	10,512,419^i^	9,877,365^i^	38,017,856^i^	^i^2,061,085
Total public expenditure on OMPs in €/capita	12.92	25.04	1.69	4.84	4.10	2.19	9.63
Average of total expenditure on OMP in €/capita in 2013 and 2014	12.26	23.50	1.41	4.43	3.68	1.76	8.92

Sources: ^a^ Federation of Austrian Social Insurance Institutions (Hauptverband der österreichischen Sozialversicherungsträger)

^b^National Institute for Health and Disability Insurance of Belgium (Institut National d'Assurance Maladie-Invalidité / Rijksinstituut voor ziekte-en invaliditeitsverzekering, INAMI / RIZIV)

^c^National Health Insurance Fund of Bulgaria, National Council on Prices and Reimbursement of Medicinal Products

^d^State Institute for Drug Control (Státní ústav pro kontrolu léčiv, SUKL)

^e^ National Health Insurance Fund Administration of Hungary (Országos Egészségbiztosítási Pénztár, OEP)

^f^National Health Fund (Narodowy Fundusz Zdrowia), Ministry of Health (Ministerstwo Zdrowia)

^g^ Health Insurance Institute of Slovenia (Zavod za zdravstveno zavarovanje Slovenije, ZZZS)

^h^ http://appsso.eurostat.ec.europa.eu/nui/show.do?dataset=ert_bil_eur_a&lang=en

^i^http://appsso.eurostat.ec.europa.eu/nui/show.do?dataset=demo_gind&lang=en

^j^Austrian data was not available on Eurostat database, the source: https://data.oecd.org/pop/population.htm

^k^Austrian data was not available on Eurostat or OECD database, the calculated number was taken from the previous year

^l^Orphan drug expenditure in hospitals not included

^m^These data was based on 10 OMs reimbursed by National Health Insurance Fund

^n^Additional drugs were identified which held orphan designation until 2013/14 with significant costs (Glivec, Ilaris, Ventavis)

^o^Data was not available at time of data request; this number assumes same total expenditure on public healthcare as in year 2013

As the Table 4 shows, average expenditures on OMPs ranged between 2.25–6.51% of the public pharmaceutical budget, and 0.44–0.96% of public healthcare expenditures in 2013–14. No data was available for Austria and Bulgaria.

**Table 4:** Total Public Expenditure on OMPs in 2013 and 2014 compared to GDP, Total Pharmaceutical and Healthcare Budget

	Austria^c^	Belgium	Bulgaria	Czech Republic	Hungary	Poland	Slovenia
2013							
Total public expenditures on OMPs (thousand €)	98,600^a^	245,000^a^	8266^a^	42,340^a^	32,262^a^	50,649^a^	16,893^a^
Total GDP (thousand €)	322,539,200^b^	391,712,000^b^	42,011,500^b^	157,741,600^b^	101,483,300^b^	394,721,100^b^	35,917,100^b^
Total public expenditures on OMPs in proportion of GDP (%)	0.03%	0.06%	0.02%	0.03%	0.03%	0.01%	0.05%
% of total pharmaceutical expenditures	3.74% ^e^	6.18% ^f^	3.07% ^g^	2.00% ^h^	3.42% ^i^	1.95% ^j^	4.65% ^k^
% of total healthcare expenditures	NA	0.91% ^f^	NA	0.4% ^h^	0.53% ^i^	0.34% ^j^	0.74% ^k^
Total pharmaceutical expenditures (thousand €) (calculated)	2,636,364	3,964,401	269,248	2,117,013	943,332	2,597,381	363,297
Total healthcare expenditures (thousand €) (calculated)	NA	26,923,077	NA	10,585,065	6,087,161	14,896,746	2,282,879
2014							
Total public expenditures on OMPs (thousand €) (Growth compared to 2013, %)	109,800 (+ 11.4%)^a^	280,000 (+ 14.3%)^a^	12,228 (+ 47.9%)^a^	50,835 (+ 20.1%)^a^	40,498 (+ 25.5%)^a^	83,341 (+ 64.5%)^a^	19,854 (+ 17.5%)^a^
Total GDP (thousand €)	330,417,600^b^	400,805,000^b^	42,762,200^b^	166,964,100^b^	104,953,300^b^	410,989,700^b^	37,332,400^b^
Total public expenditures on OMPs in proportion of GDP (%)	0.03%	0.07%	0.03%	0.03%	0.04%	0.02%	0.05%
% of total pharmaceutical expenditures	3.94%^e^	6.84% ^f^	4.15% ^g^	2.50% ^h^	4.25% ^i^	3.2% ^j^	5.47% ^k^
% of total healthcare expenditures	NA	1.01% ^f^	NA	0.60% ^h^	0.66% ^i^	0.54% ^j^	0.84% ^k^
Total pharmaceutical expenditures (thousand €) (calculated)	2,786,802	4,093,567	294,654	2,033,406	952,883	2,604,432	362,956
Total healthcare expenditures (thousand €) (calculated)	NA	27,722,772	NA	8,472,525	6,135,990	15,433,670	2,363,538
Total public expenditures on OMPs in proportion of GDP (%) in 2013	0.03%	0.06%	0.02%	0.03%	0.03%	0.01%	0.05%
Total public expenditures on OMPs in proportion of GDP (%) in 2014	0.03%	0.07%	0.03%	0.03%	0.04%	0.02%	0.05%
Average	0.03%	0.07%	0.02%	0.03%	0.04%	0.02%	0.05%
Total public expenditure on OMPs as a proportion of total public *pharmaceutical* expenditures in 2013	3.74%	6.18%	3.07%	2.00%	3.42%	1.95%	4.65%
Total public expenditure on OMPs as a proportion of total public *pharmaceutical* expenditures in 2014	3.94%	6.84%	4.15%	2.50%^d^	4.25%	3.20%	5.47%
Average	3.84%	6.51%	3.61%	2.25%	3.84%	2.58%	5.06%
Total public expenditure on OMPs as a proportion of total public *healthcare* expenditures in 2013	NA	0.91%	NA	0.40%	0.53%	0.34%	0.74%
Total public expenditure on OMPs as a proportion of total public *healthcare* expenditures in 2014	NA	1.01%	NA	0.60%^d^	0.66%	0.54%	0.84%
Average	NA	0.96%	NA	0.50%	0.60%	0.44%	0.79%

Sources: ^a^ Data was given from Table 1

^b^http://ec.europa.eu/eurostat/tgm/refreshTableAction.do?tab=table&plugin=1&pcode=tec00001&language=en

^c^Orphan drug expenditure in hospitals not included

^d^Data was not available at time of data request. this number assumes same total expenditure on public healthcare as in year 2013

^e^Federation of Austrian Social Insurance Institutions (Hauptverband der österreichischen Sozialversicherungsträger)

^f^National Institute for Health and Disability Insurance of Belgium (Institut National d'Assurance Maladie-Invalidité / Rijksinstituut voor ziekte-en invaliditeitsverzekering, INAMI / RIZIV)

^g^National Health Insurance Fund of Bulgaria, National Council on Prices and Reimbursement of Medicinal Products

^h^State Institute for Drug Control (Státní ústav pro kontrolu léčiv, SUKL)

^i^National Health Insurance Fund Administration of Hungary (Országos Egészségbiztosítási Pénztár, OEP)

^j^National Health Fund (Narodowy Fundusz Zdrowia), Ministry of Health (Ministerstwo Zdrowia)

^k^Health Insurance Institute of Slovenia (Zavod za zdravstveno zavarovanje Slovenije, ZZZS)

Data on the public expenditure of the 10 selected OMPs in different therapeutic areas were not available in France and Austria. Compared to the average spending of participant countries, Belgium and Slovenia had significantly higher spending, whilst spending in Bulgaria and Poland was far below the average. (See Table 5.)

**Table 5:** Public Spending on 10 Selected Orphan Medicinal Products in 6 EU Countries in 2013–14

	Average spending per 100,000 inhabitants in 2013–14	Ratio of spending compared to the average of 6 countries
Belgium	281,878 €	2,15
Bulgaria	34,586 €	0,26
Czech Republic	115,187 €	0,88
Hungary	83,097 €	0,63
Poland	51,591 €	0,39
Slovenia	219,926 €	1,68

We made a similar calculation for those three OMPs that were accessible for patients with public reimbursement in the countries in 2013–14 (see Table 6). According to the Hungarian regulation, financial data of some OMPs among the indicators were allowed to published only as summarized amounts, therefore we were not able to separate the annual total expenditures of these OMPs, thus we had to ignore the Hungarian data.

**Table 6:** Public Spending on 3 Orphan Medicinal Products with Reimbursement in 5 EU Countries in 2013–14

	Average spending per 100,000 inhabitants in 2013–14	Ratio of spending compared to the average of 5 countries
Belgium	94,744 €	1,39
Bulgaria	33,387 €	0,49
Czech Republic	80,526 €	1,18
Poland	51,565 €	0,76
Slovenia	80,699 €	1,18

Based on the ratio of country specific spending compared to the average spending of participant countries (see Table 5 and Table 6) similar tendency can be observed; wealthier countries spend more per capita on ODs than lower-income countries.

## Discussion

According to our data, accessibility to OMPs is associated with the economic status of the member state (MS) [22, 27]. In Austria, Belgium and France more OMPs were accessible for patients with public reimbursement than in Bulgaria, Czech Republic, Hungary, and Poland indicating an equity gap between Eastern and Western Europe. Slovenia was in between Western-European and CEE countries.

The reimbursement categories of OMPs are related to different decision criteria. Although market access criteria of OMPs with standard reimbursement listing through retail pharmacies may not be as strong as criteria for pharmaceuticals in common diseases, OMPs with such reimbursement status at least go through a centralised evaluation procedure and price negotiation in the MSs. Evaluation criteria and price negotiation for OMPs with standard reimbursement in hospitals only may be less sophisticated in some countries compared to OMPs on the outpatient reimbursement list.

In Belgium and France other than standard reimbursement techniques do not exist. Special individual reimbursement might be available only for children (< 18 years) in Bulgaria. However, the application of special reimbursement for individual patients cannot be associated with the economic status of participant countries.

There are substantial differences in the total public expenditure on OMPs per capita in participant countries. The absolute spending is clearly associated with the economic status of countries. If we assume a narrow price corridor across countries due to the widely used external price referencing system, confidential discounts, unequal spending translates to inequity in access to OMPs. Interestingly, the spending on OMPs as a proportion of GDP, public pharmaceutical and healthcare expenditure was not higher in lower-income countries compared to those with higher-income, which also indicates substantial differences in patient access to OMPs in favour of higher-income countries. However, we must emphasize that utilisation of OMPs highly depends on many other factors, including the prevalence of RDs or the availability of diagnostic facilities.

European collaboration is a crucial need to improve equal access to OMPs across the European Union. Regarding the registration and health technology assessment step, existing networks, initiatives and proposals - such EUnetHTA [28]; EU proposal on HTA [29] - have a predominant role to exchange and collect information, and summarize knowledge in order to create one therapeutic, scientific compilation report to support the centralized procedure of the marketing authorization; and to facilitate the decision-making process with semi-qualitative transparent value matrix as the MOCA project recommended or with the framework provided by the multi-criteria decision analysis model [30]*.*

It should maintain bilateral connections for the national HTA authorities - to participate in the information summarization and to provide a well-informed basis for the national assessment procedure [31, 32]*.*

The central regulatory body should require and provide the compilation report accessible at the time of marketing authorization and towards, as well as the permanent mapping and announcement of unmet needs of MSs.

The generalizability of the findings may be limited due to several reasons. First of all, it should be emphasized that the orphan status of medicines is flexible and can change over time. We have not evaluated the availability of other funding channels, including early access programs, donations, and compassionate use. Finally, we did not have access to all of the data in every participant country.

## Conclusions

European policymakers pay special attention to positively discriminate patients with rare diseases. There are international policy tools to facilitate the research and development of orphan medicinal products, and payers in some countries may apply special criteria to approve the reimbursement of OMPs. Consequently we can state that equity in OM access for patients with rare diseases is an important policy objective in each member state of the European Union. However, our study indicates serious inequity in access across EU member states, which requires additional research and consequently harmonised policy actions at the European level.

Our research could not prove that the public funding of OMPs would be a greater burden to the lower-income countries, mainly because these countries manage the sustainability of public healthcare funding by limiting patient access to high cost therapies.

## Additional file

Additional file 1: The Map for Funding Orphan Medicinal Products in 8 EU Member States in 2015. (DOCX 63 kb)

## Abbreviations

CEE: Central-, and Eastern European countries
CHMP: Committee for Medicinal Products for Human Use
COMP: Committee for Orphan Medicinal Products
EMA: European Medicines Agency
EPAR: European Public Assessment Report
ERT: Enzyme Replacement Therapy
EU: European Union
FDA: Food and Drug Administration of the United States of America
HAS: (Haute Autorité de Santé) - the French National Authority for Health
HCP: HealthCare Provider
HTA: Health Technology Assessment
HVSVT (in German: Hauptverband der Österreichischen Sozialversicherungsträger): Federation of Austrian Social Insurance Institutions
ICD: International Classification of Diseases
INAMI (Institut National d'Assurance Maladie-Invalidité) – RIZIV (Rijksinstituut voor Ziekte- en Invaliditeitsverzekering): National Institute for Health and Disability Insurance of Belgium
MS: Member State
NHIF: National Health Insurance Fund
NHIFA: National Health Insurance Fund Administration of Hungary (in Hungarian: Országos Egészségbiztosítási Pénztár – OEP)
NPRD: National Plan for Rare Diseases
OECD: Organisation for Economic Co-operation and Development
OMP(s): Orphan Medicinal Product(s)
PDL: Positive Drug List
R&D: Research and Development
RDs: Rare Diseases
SÚKL (in Czech: Státní ústav pro kontrolu léčiv): State Institute for Drug Control
WHO: World Health Organization
ZZZS (in Slovenian: Zavod za Zdravstveno Zavarovanje Slovenije): The Health Insurance Institute of Slovenia
