# Supplementary material for: The European challenges of funding orphan medicinal products
Source: Orphanet J Rare Dis. 2018 Nov 6;13:184. doi: 10.1186/s13023-018-0927-y (PMC6219168; doi:10.1186/s13023-018-0927-y)
Supplement: Supplementary file 1 — The Map for Funding Orphan Medicinal Products in 8 EU Member States in 2015. (DOCX 63 kb) [file 13023_2018_927_MOESM1_ESM.docx]

The Map for Funding Orphan Medicinal Products in 8 EU Member States in 2015

Sources:

Austria: Federation of Austrian Social Insurance Institutions (Hauptverband der österreichischen Sozialversicherungsträger)

Belgium: National Institute for Health and Disability Insurance of Belgium (Institut National d'Assurance Maladie-Invalidité / Rijksinstituut voor ziekte-en invaliditeitsverzekering, INAMI / RIZIV)

Bulgaria: National Health Insurance Fund of Bulgaria, National Council on Prices and Reimbursement of Medicinal Products

Czech Republic: State Institute for Drug Control (Státní ústav pro kontrolu léčiv, SUKL)

France: French National Authority for Health (Haute Autorité de Sante, HAS)

Hungary: National Health Insurance Fund Administration of Hungary (Országos Egészségbiztosítási Pénztár, OEP)

Poland: National Health Fund (Narodowy Fundusz Zdrowia), Ministry of Health (Ministerstwo Zdrowia)

Slovenia: Health Insurance Institute of Slovenia (Zavod za zdravstveno zavarovanje Slovenije, ZZZS)
